# Mosquito immune responses to *Plasmodium* parasites that limit malaria transmission

**DOI:** 10.1007/s00018-025-05667-z

**Published:** 2025-04-07

**Authors:** Ana Beatriz F. Barletta, Carolina Barillas-Mury, Alvaro Molina-Cruz

**Affiliations:** Laboratory of Malaria and Vector Research, National Institutes of Allergy and Infectious Diseases, National Institutes of Health, Rockville, Maryland, 20852 USA

**Keywords:** Mosquito immune response, Mosquito, Plasmodium, Malaria transmission, Plasmodium mosquito interaction

## Abstract

The mosquito immune system is a major barrier that malaria parasites must overcome for their successful development and disease transmission. At each developmental stage in the vector, *Plasmodium* parasites can be potentially targeted by the mosquito innate immune responses, which involve epithelial, humoral, and cellular components. The immune response to *Plasmodium* ookinetes can be powerful and some of the underlying effector mechanisms are well characterized. However, the defense responses to oocysts and sporozoites appear to be less effective and are less well understood. *Plasmodium* parasites are under constant pressure to avoid elimination by evading and/or manipulating the mosquito immune system. Understanding the intricate interaction between *Plasmodium* parasites and the mosquito immune system is fundamental to understand the epidemiology of malaria transmission and to devise innovative control strategies.

## Introduction

Malaria, a life-threatening parasitic disease, is caused by *Plasmodium* parasites that are transmitted by more than 70 different *Anopheles* mosquito species throughout the world [1]. Despite the significant advances in disease control in the last 20 years, malaria still results in more than 200 million cases and 600,000 deaths annually [2]. Although five parasite species infect humans (*Plasmodium falciparum*,* Plasmodium vivax*,* Plasmodium ovale*,* Plasmodium malariae and Plasmodium knowlesi*), *P. falciparum* is responsible for most of the morbidity and mortality, primarily of young children in sub-Saharan Africa. The main strategies to control malaria include the use of insecticide-impregnated bed nets, indoor residual spraying, massive administration of antimalarial drugs, diagnosis using rapid diagnostic tests followed by treatment and, more recently, a partially effective vaccine [2]. The effectiveness of some of these methods has decreased over time due to the emergence of insecticide-resistant mosquitoes and of drug-resistant parasites, making it critical to develop new control strategies.

**Fig. 1:**
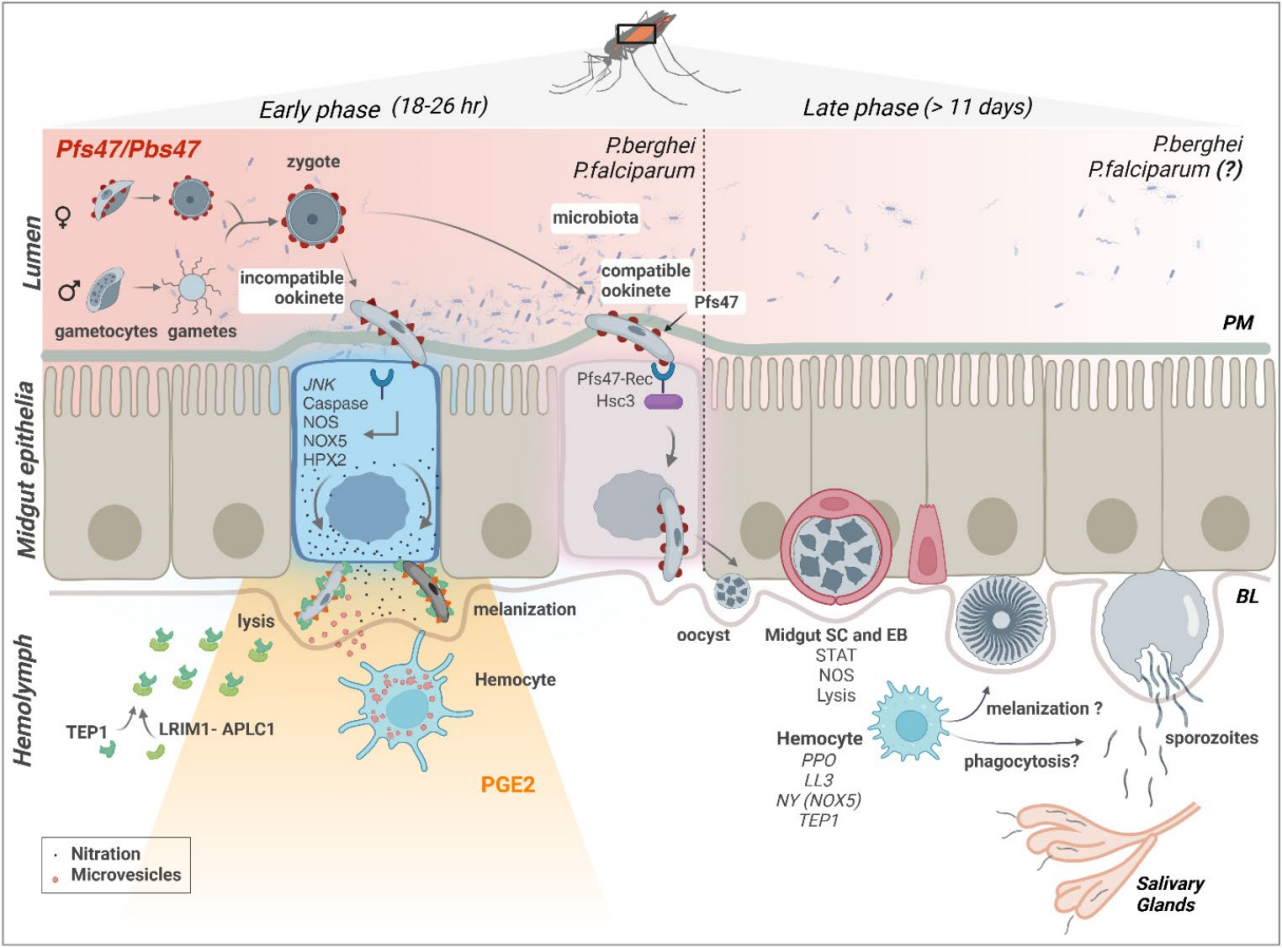
Malaria parasite development in the mosquito and interactions with the mosquito immune system. The figure shows the working model of the early and late phase humoral and cellular components of the mosquito immune system that can limit *Plasmodium* infection. Sexually differentiated *Plasmodium* gametocytes taken up in a blood meal, undergo a complex developmental cycle in the mosquito midgut as the ingested blood is digested and the microbiota proliferates. Ookinetes with a Pfs47 haplotype compatible with a given mosquito vector evade the early phase immune response of the mosquito’s innate immune system and develop into oocysts. In contrast, those that lack P47 or express an incompatible or partially compatible haplotype trigger caspase 2-mediated apoptosis in the invaded cell that involves activation of the Janus Kinase pathway (JNK), which mediates nitration of the invaded cell and the adjacent basal lamina. Disruption of the midgut peritrophic matrix (PM) exposes midgut epithelial cells to the gut microbiota and triggers prostaglandin E2 (PGE2) release. PGE2 attracts hemocytes to the invaded cell, and they release microvesicles (HDMV) when they encounter a nitrated basal lamina (BL), which in turn leads to thioester protein 1 (TEP1) binding that result in parasite elimination by lysis and/or melanization. TEP1 is stabilized in the hemolymph by interacting with a leucine rich repeat proteins LRIM1/ APLC1 complex. The ookinetes that manage to successfully reach the basal lamina, differentiate into early oocysts and develop under the basal lamina (BL) for at least 11 days before rupture. During development, *P. berghei* oocysts are susceptible to elimination by midgut stem cells (SC) and enteroblasts (EB). SC divide and semi-differentiate around the oocysts, limiting their growth and promoting lysis. This response is mediated by the STAT pathway and NOS (Nitric Oxide Synthase). Furthermore, *P. yoelii* oocysts in which the PEXEL motifs of CS are mutated trigger a strong TEP1-mediated melanization response that involved NOX5 and LL3 expression, *Toll* signaling and hemocyte nitration. When oocysts rupture, sporozoites are released into the hemolymph, where they travel until they reach the salivary glands and invade them. Question mark indicates the lack of information regarding late phase immune resposens against P. falciparum.

It is attractive to target the parasite in the mosquito, due to the natural bottleneck in the number of parasites that occurs during the complex development in the vector. Female mosquitos become infected when they ingest a blood meal containing *Plasmodium* gametocytes (Fig. 1). These sexually differentiated parasites transform within minutes into male and female gametes that fuse to form a fertilized zygote in the midgut lumen. It takes about a day for the zygote to develop into a motile ookinete that invades and traverses the midgut epithelium. Only a few ookinetes succeed in reaching the midgut basal lamina and transform into oocysts, which undergo sporogony and multiply for two weeks before releasing thousands of sporozoites into the mosquito hemolymph. Sporozoites invade the salivary gland and are transmitted when an infected mosquito bites a new vertebrate host.

The development of *Plasmodium* in mosquitoes is limited by biotic and environmental factors [3], including environmental temperature and humidity, the mosquito microbiota, and the mosquito immune response to the parasite. Like other insects, anopheline mosquitoes have an effective innate immune system with humoral and cellular components that can eliminate microbial pathogens by melanotic encapsulation, lysis or phagocytosis [4]. Several signaling pathways, such as the Toll, Imd, Jak/STAT and JNK pathways, regulate mosquito immune responses [5], and the complement-like system is a well-characterized effector of defense responses that target bacteria and the *Plasmodium* ookinete stage [6, 7]. Although mosquitoes lack an acquired immune system, they can enhance their immune response to pathogens they have previously encountered through an immune priming response [8].

The mosquito innate immune system can limit *Plasmodium* infection by activating effector mechanisms that target different developmental stages of the parasite. *Plasmodium* species adapted to a given vector can evade the mosquito immune system and are therefore compatible with the vector. However, some *Plasmodium* strains or species are partially compatible or incompatible with a specific vector and trigger immune responses that eliminate the parasite [9]. Consequently, the immune response of a given anopheline mosquito species (or strain) against different malaria parasite species (or strains) may differ drastically, according to the degree of parasite/vector compatibility [10]. The interaction of partially compatible vector/parasite combinations such as the infection of *An. gambiae*, a major vector of *P. falciparum* malaria in Africa, with mouse *Plasmodium berghei* parasites, have been instrumental to uncover immune responses that the mosquito can potentially mount against a parasite [7]. The elucidation of antimalarial responses to *P. berghei* made it possible to begin to address the question of how parasites well adapted to their sympatric mosquito vectors, such as African *P. falciparum* parasites that infect *An. gambiae*, avoid triggering these responses, and how *Plasmodium* immune evasion affects malaria transmission.

Here we review recent progress and remaining gaps in our understanding of the interactions between the mosquito immune system and malaria parasites. We sequentially explore the mosquito defense responses that target ookinetes, oocysts and sporozoites, as the parasite develops in the insect vector.

### Early phase immunity: mosquito immune response to ookinetes

*Plasmodium* zygotes develop into motile ookinetes in the harsh and toxic environment of a blood meal undergoing digestion in the midgut lumen. Blood feeding triggers the secretion of mucins, and of a chitinous material by the mosquito gut that forms the peritrophic matrix, which surrounds the blood meal and prevents direct contact of the gut microbiota with the epithelium [11]. *Plasmodium* ookinetes secrete a chitinase enzyme that allows them to disrupt and cross the peritrophic matrix to reach the apical surface of the epithelium [12]. Blood digestion also results in extensive proliferation of the gut microbiota, which can directly limit parasite survival by releasing products toxic to the parasite or indirectly, by activating mosquito immune responses detrimental to the parasite [13].

The current working model of defense responses to ookinetes is mostly based on studies with *P. berghei* infection of *An. gambiae.* In this partially compatible model system, ookinetes cause irreversible damage to the midgut cells they invade, which ultimately leads to activation of the mosquito complement-like system and elimination of most parasites. Invaded cells activate a caspase-mediated apoptotic response that involves induction of nitric oxide synthetase (NOS) expression, and of JNK-mediated induction of heme peroxidase 2 (HPx2) and nicotinamide adenine nucleotide phosphate oxidase 5 (NOX5) expression in the invaded cell. These enzymes mediate a strong nitration reaction in the apoptotic cell and the adjacent basal lamina [14–17] (Fig. 1).

Disruption of the peritrophic matrix leads to direct contact of the microbiota with the midgut cells, which respond by producing and secreting prostaglandin E2 (PGE2) into the hemolymph [18]. PGE2 is a chemoattractant for hemocytes, both in vitro [18] and in vivo [18], and PGE2 release attracts hemocytes to the basal lamina. Hemocytes that come in contact with a nitrated basal lamina release microvesicles in proximity to the invaded cell, which are essential for effective activation of thioester containing protein 1 (TEP1), a key effector of the mosquito complement-like system [19].

TEP1 is functionally similar to the vertebrate complement protein C3 and is constitutively present in the hemolymph as a cleaved form (TEP1cut) in a stable complex with two leucine-rich repeat proteins, LRIM1 and APL1C. Activation of TEP1cut involves release from the LRIM1/APL1C complex [20, 21]. A non-catalytic serine protease, SPCLIP1, is a key regulator of the complement-like pathway required for accumulation of TEP1 on microbial surfaces. It has been proposed that SPCLIP1 is a co-factor that locally activates a serine protease which cleaves an abundant inactive uncut full-length TEP1 (TEP1-F) [22]. The TEP1 convertase remains to be identified, but one can envision that hemocyte-derived microvesicles may deliver this enzyme (or cofactors), propagating local activation of TEP1-F to TEP1cut that covers the ookinete surface and damages the parasite [22]. Conversely, the clip-domain serine protease CLIPA2 down regulates TEP1 cleavage [23].

Previous exposure to *Plasmodium* infection enhances the mosquito immune response to subsequent infections through a priming response that requires ookinete midgut invasion in the presence of the gut microbiota [24]. Priming results in a constitutive increase in the proportion of granulocytes. Furthermore, silencing Cactus, a suppressor of Toll signaling, results in total elimination of the parasite [25]. This overactivation of *Toll* signaling also dramatically increases the proportion of megacytes, a subpopulation of very plastic giant granulocytes, from 5 to 80% of granulocytes [26]. A unique molecular marker for megacytes demonstrated that the number of these cells associated with the midgut increases dramatically in response to ookinete invasion [26] and greatly increases local microvesicle release [19]. These studies highlight the importance of hemocytes in early immune responses to the parasite [18, 19, 26].

The genetically selected *An. gambiae* refractory L35 strain eliminates *P. cynomolgi* and almost all *Plasmodium* parasite species tested, including *P. falciparum* 7G8 from South America, through a melanotic response that involves TEP1 activation. In contrast, *P. falciparum* strains from Africa [27], such as GB4, evade the mosquito complement system and complete their development without being melanized [27, 28]. A combination of quantitative trait loci (QTL) analysis, linkage group selection and functional genetics were used to identify Pfs47 as the gene coding for a surface protein that makes ookinetes “invisible” to the mosquito immune system [28]. Disruption of Pfs47 in African type *P. falciparum* led to parasite elimination that was prevented by silencing TEP1 expression [28]. A direct comparison of the mosquito immune responses to infection with wild-type (*wt*) and Pfs47 knockout (*ko*) *P. falciparum* demonstrated that parasites that lack Pfs47 expression trigger immune responses very similar to those of *P. berghei*, which involve JNK activation and caspase-mediated apoptosis of ookinete-invaded cells that induces NOX5 and HPx2 expression, followed by nitration, local microvesicle release by hemocytes, TEP1 activation and ookinete lysis (Fig. 1) [29, 30].

Pfs47 is a polymorphic gene whose haplotypes present a marked geographic population structure between continents [9, 31]. Side by side infections of *P. falciparum* lines from different continents with malaria vectors from Africa (*An. gambiae*), Asia (*An. dirus*) and Central/South America (*An. albimanus*), showed that parasites are more compatible with mosquitoes from the same geographic region [9]. Furthermore, disrupting the complement-like system by silencing TEP1 or LRIM1 (a stabilizer of TEP1) prevented parasite elimination in infections with incompatible combinations and switching the Pfs47 of African NF54 parasites with haplotypes present in other continents changed their compatibility with the different vector species [9]. Four amino acids located in Pfs47-Domain 2, between the two cysteines, are major determinants of vector compatibility [32].

Based on these observations, the “lock and key” model was proposed, in which ookinetes with a compatible Pfs47 on their surface (“the key”) disarm the mosquito immune response by interacting with a receptor (“the lock”) in the mosquito midgut [29, 33]. The Pfs47 Receptor (P47Rec) was identified as a DM9 domain protein present in the sub microvillar region of mosquito epithelial cells. Recombinant P47Rec binds to recombinant Pfs47 with high affinity and midgut expression of P47Rec is required for parasites to evade mosquito immunity [29]. Pfs47 in African type NF54 parasites prevents caspase-mediated apoptosis of the invaded cell in *An. gambiae* (G3) [29] by interacting with P47Rec, which in turn binds to Hsc70-3, a heat shock protein that prevents caspase S2-dependent apoptosis and accelerates the extrusion of invaded cells [30]. Pfs47 knockout NF54 ookinetes elicit a very similar immune response in An. *gambiae* to that of *P. berghei*, with activation of caspases [29, 30], epithelial nitration [29], extensive hemocyte microvesicle release and parasite lysis [30]. Studies on the potential of Pfs47 as a transmission blocking vaccine target showed that antibodies against a 46 AA region of Pfs47 strongly reduced mosquito infection by preventing ookinete development [34].

*P. falciparum* Pfs16, a parasitophorous membrane protein required for the optimal formation of gametocytes [35], also reduces the mosquito immune response to the ookinete. Pfs16 appears to act by down regulating the activity of caspase 3/7 in the midgut and antibodies against Pfs16 have transmission blocking activity [36, 37]. A *P. berghei* mutagenesis screen identified “Infection of the Mosquito Midgut Screen 43” (PIMMS43) as a necessary gene for immune evasion, as ookinetes lacking PIMMS43 protein failed to infect the midgut [38]. However, this gene has additional functions besides immune evasion, because although TEP1 silencing rescued the ookinete stage, no viable sporozoites develop within the oocysts [38].

Overactivation of the IMD immune pathway by silencing the suppressor Caspar reduced *P. falciparum* infection in *An. gambiae*, *An. stephensi* and *An. albimanus* [39, 40]. Furthermore, immune-enhanced transgenic *An. stephensi* mosquito lines expressing additional copies the mosquito NF-kB Rel2 under blood meal-inducible promoters driving expression in the midgut or fat body, significantly reduced *P. falciparum* infection through activation of the mosquito complement system and enhanced resistance to bacterial challenge [41]. Melanotic encapsulation [42] can be the final effector of the mosquito anti-plasmodial immune response or a mechanism for disposing of dead ookinetes [43]. Melanization is catalyzed by phenoloxidases whose proteolytic activation is regulated by serine proteinase cascades, including clip domain proteinases (CLIPs). Several CLIPs are positive regulators of melanization (SCLIP1, CLIPA8, CLIPA28, CLIPB8, CLIPB4, CLIPB14, CLIPB10, CLIPB17 and CLIPC9) [22, 43–45] while others are negative regulators (CLIPA2 and CLIPA14) [23, 42]. Members of the Serpin family of serine proteinase inhibitors also regulate the melanization pathway [46]. For example, serpin-2 (SRPN2) inhibits CLIPB4, CLIPB10 and CLIPB9 [47]. Furthermore, *Plasmodium* ookinetes avoid melanization by coopting C-type lectins CTL4 and CTLMA2 onto their surface, by a mechanism that is not yet understood [48–50].

### Late phase immunity: mosquito immune response to oocysts and sporozoites

Late phase immune responses against *Plasmodium* oocysts and sporozoites are less well understood than early mechanisms of parasite elimination. Ookinetes that reach the midgut basal lamina differentiate into oocyst, a stage in which each parasite forms a capsule, multiplies extensively, and grows for a period of at least 11 days. Mature oocysts rupture and release thousands of sporozoites into the mosquito hemolymph. Oocysts are considered a “quiet” developmental stage, in which the capsule and bound mosquito proteins (laminin, lysozyme c-1, and matrix metalloprotease 1) allow parasites to develop undetected by the mosquito immune system [51, 52]. However, there is growing evidence of specific mosquito immune responses that target oocysts.

Two different types of immune responses to developing oocysts have been described, those mediated by midgut progenitor cells (stem cells and enteroblasts) [53] and hemocyte-mediated responses [52]. Midgut stem cells maintain the integrity of the tissue by proliferating and dividing asymmetrically to generate two cells: an enteroblast that will differentiate to replace a damaged cell and a new stem cell that will remain undifferentiated to maintain the pool of stem cells in the tissue [54–56]. Recent studies revealed that *P. berghei* oocysts trigger extensive proliferation of midgut stem cells and partially differentiated enteroblasts in *Anopheles stephensi* [53]. Enteroblasts surround the oocysts and limit their survival (Fig. 1). Intravital microscopy revealed that enteroblasts project filopodia-like structures that come in contact with the oocyst surface and trigger the release of their cytoplasm into the midgut lumen as the oocyst is eliminated [53]. Activation of the STAT pathway by silencing its repressor SOCs increases proliferation and differentiation of stem cells into enteroblasts and decreases oocyst survival [53]. The number of oocysts present 2 days post-infection decreases five-fold by day 8 post-infection, indicating that about 80% of early oocysts are eliminated. Furthermore, this decrease in oocyst numbers between days 2 and 8 post-infection is not observed when STAT-A is silenced [57]. The elimination of oocysts when STAT signaling is overactivated by silencing SOCS, a suppressor of STAT, is mediated by nitric oxide synthase (NOS) [57], but how NO affects enteroblasts and oocysts is not known. Several questions remain regarding the mechanism of oocyst elimination. The presence and persistent growth of *P. berghei* oocysts exerts mechanical pressure on epithelial cells and results in extensive remodeling of the midgut architecture. However, it is not clear if midgut stem cell proliferation is triggered in response to mechanical pressure on neighboring cells or whether oocysts release some factor(s) that activate stem cells. When ookinetes reach the basal lamina and transform into oocysts, they do not co-localize with midgut stem cells [53], suggesting that midgut progenitors proliferate and migrate towards the oocysts. It remains unclear whether NO release could be a signal that directs midgut stem cell migration towards the oocysts.

The transcription factor LL3 controls hemocyte differentiation and reducing expression by dsRNA silencing increases oocyst survival [52], suggesting that hemocytes may also target *Plasmodium* oocysts. Interesting, LL3 is highly expressed in megacytes, which release microvesicles critical for complement-mediated elimination of ookinetes [26, 58]. However, it is not clear if they are also active against oocysts. Furthermore, reducing expression of three different prophenoloxidases (PPO2, PPO3 and PPO9), enzymes involved in melanization that are highly expressed in oenocytoids, also increase oocyst survival through a TEP1-independent mechanism [59]. However, it is not clear how PPOs mediate parasite lysis that does not involve melanization.

The circumsporozoite protein (CS) is the most abundant protein on the sporozotie surface and a major vaccine target to prevent malaria infection. Wild type *P. berghei* and *P. yoelii* oocysts are not melanized by *An. stephensi* mosquitoes, but mature oocysts from parasites in which the PEXEL motifs of CS were mutated triggered a strong melanization response to both species that dramatically reduced the number of sporozoites that reached the salivary glands [60]. Parasite elimination was mediated by NOX5-mediated hemocyte nitration, with TEP1 binding as a critical effector mechanism that required activation of *Toll* signaling [60]. The observation that only mature oocysts were melanized suggests that contact of CS with mosquito hemolymph and hemocytes is necessary to activate this defense response. It is very interesting that besides the role of NOS and NOX5 in epithelial nitration following ookinete invasion [14], mutant parasites activated nitration in hemocytes, and that this also leads to TEP1-mediated parasite elimination.

Moreover, mosquitoes that were first infected with CS-mutant parasites and then infected with wild-type *Plasmodium*, acquired the ability to also eliminate wild-type parasites from the second infection, while a previous infection with wild-type parasites did not trigger this response [60], indicating that exposure to mutant parasites activated a novel mechanism of immune priming. These findings suggest that wild-type CS allows parasite development without immune recognition, but this mechanism fails once the system is primed by mutant parasites. The potential participation of other hemocytes, such as oenocytoids, and the PPOs involved in melanization of the mutant oocysts remain unknown. Immune mechanisms described above were characterized using murine *Plasmodium* species. Little is known about the mosquito immune responses to *P. falciparum* oocysts and whether they differ from those elicited by *P. berghei.* However, a previous study showed that disrupting STAT signaling in *An. gambiae* resulted in a modest increase in the number of *P. falciparum* oocysts [57].

When oocysts rupture, sporozoites are released and come in contact with humoral immune effectors in the hemolymph and with circulating hemocytes. Phagocytosis of sporozoites by hemocytes has been reported, but it is an infrequent event [61]. The observation that hemocytes are more efficient in taking up bacteria than sporozoites suggested that sporozoites may actively avoid phagocytosis in their transit to the salivary glands [61]. In fact, a mechanism was recently identified by which *Plasmodium* sporozoites evade elimination by the mosquito immune system through Glutaminyl Cyclase (QC)-mediated modification of sporozoite proteins. QC is an enzyme that catalyzes the post-translational modification of N-terminal Gln or Glu AA residues to pyroglutamate by forming a lactam ring [62]. In humans, QC is known to modify CD47, a protein highly expressed in human cancer cells, and this modification helps malignant cells evade immune surveillance by making them resistant to phagocytosis [63]. The importance of *Plasmodium* QC to prevent elimination of sporozoites by circulating hemocytes was explored by disrupting the QC gene in *P. berghei* and *P. falciparum*. Lack of QC expression leads to melanization and phagocytosis of sporozoites as they encounter the mosquito hemolymph. *P. berghei* parasites expressing a catalytically dead QC were also eliminated, confirming that the enzymatic activity of QC is required for *Plasmodium* to avoid melanization and phagocytosis. Furthermore, CS was shown to be an important QC substrate, as N-terminal pyroglutamate modification of CS by QC was necessary for the parasite to evade mosquito immunity [62]. Co-silencing of CLIPA14 and CLIPA2, both negative regulators of melanization in *An. gambiae*, also results in a significant increase in TEP-1 mediated oocyst melanization in both *P. berghei* and *P. falciparum* as the oocysts rupture, suggesting that these regulators normally raise the threshold to activate the melanization cascade [64].

## Concluding remarks

Mosquito epithelial, humoral and cellular defense responses can be a significant barrier to *Plasmodium* infection and malaria transmission. The early phase of the mosquito immune response against ookinetes can be very effective against incompatible parasites and is mainly mediated by the complement-like system. However, some open questions remain, such as, the nature of the complex that eliminates ookinetes after TEP1 binding on the surface of the parasite, the mechanism by which pathogens are recognized by the TEP1cut/LRIM1/APL1, and how this leads to the release of active TEP1cut from this stabilizing complex.

The late phase immune responses that target oocysts and sporozoites, appear to be less effective than ookinete elimination and our understanding of these mechanism is more limited. There are some similarities between early and late immune responses, such as the involvement of nitration in ookinete-invaded midgut cells and in hemocytes of mosquitoes infected with parasites in which the CS surface protein in the sporozoite surface has been mutated. Both reactions are also mediated by NOX5 and trigger TEP1 binding to the parasite’s surface. Interestingly, infection with a mutant CS confers a more effective response against subsequent infections with wild-type parasites. This phenomenon mirrors the innate immune priming response in which contact of the gut microbiota with the epithelia during ookinete invasion leads to long-lasting changes in hemocyte populations that enhance the response to a second infection. This suggests that there may be some coordination between early and late phase mechanisms of immune priming, where an early response to an incompatible ookinete may also enhance the immune response to oocysts or sporozoites.

The characterization of mosquito immune defenses using incompatible animal models of malaria transmission has made it possible to elucidate the mechanism that allow *P. falciparum* parasites to be effectively transmitted by sympatric anophelines, and translational strategies to prevent disease transmission must address these evasion mechanisms. *Plasmodium* populations are under constant selective pressure to avoid detection and elimination by the mosquito immune system. Nevertheless, a parasite that has adapted well to a given mosquito species, may remain incompatible to evolutionary distant anophelines. When humans infected with a parasite move to a new geographic region where the mosquito vectors are different, only those parasites that can avoid elimination by mosquito immune defenses can establish a new local disease transmission cycle. The differences in compatibility of parasite populations to different vectors is an important selective force that constantly shapes the population structure of *Plasmodium* and understanding the molecular mechanisms involved brings new insights into the epidemiology of malaria transmission.

## Data Availability

Not applicable.
